# Chromosomal Organization: Mingling with the Neighbors

**DOI:** 10.1371/journal.pbio.0040155

**Published:** 2006-05-16

**Authors:** Jacob A Aten, Roland Kanaar

## Abstract

Jacob Aten and Roland Kanaar highlight recent advances in understanding the physical organization of chromosomes in the nucleus.

Chromosomes, the packaged DNA carriers of heredity and instructions for proper cell functioning, undergo dramatic morphological transformations during the cell-division cycle. In metaphase, which includes the alignment of chromosomes before their separation between the two daughter cells, the 46 chromosomes in a human cell are condensed to such a degree that they can be observed by light microscopy as clearly separate individual entities. In cells that have entered the subsequent interphase, the chromosomes partially decondense into chromosome territories [ 1]. The information contained in chromosomes is retrieved and acted upon in their (partly) decondensed state. A precise understanding of how decondensed interphase chromosomes interact is important because close contact between and within chromosomes has implications for such fundamental processes as transcription and DNA damage repair; chromosome association can influence gene expression [ 2, 3], and misrepair of DNA double-strand breaks can promote genome instability in the form of chromosome translocations [ 4, 5].

A key issue in chromosome biology is to determine if, and if so to what extent, different chromosomes interact in the nucleus. A number of arrangements are possible ( Figure 1). At one end, there is a complete lack of interaction: chromosomes may be contiguous without intermingling, or they may be separated by interchromatin domains. Interchromatin domains are nuclear areas mostly void of chromatin where important chromosomal transactions such as transcription, pre-mRNA splicing, DNA damage repair, and DNA replication occur. At the other extreme, chromatin loops of different chromosomes can freely intermingle, leading to a situation where the borders between chromosome territories and chromosome subdomains are no longer clearly defined.

**Figure 1:**
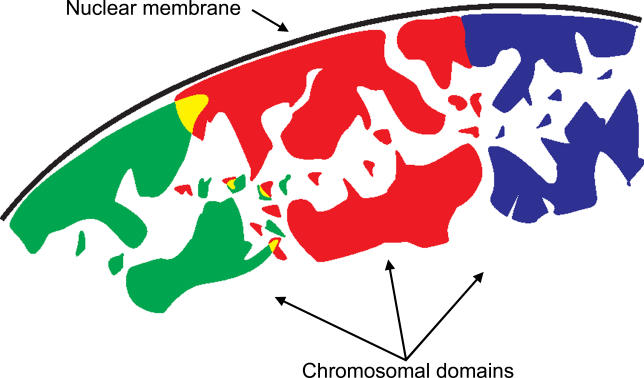
Schematic Illustration of Possible Neighborhood Organization Modes of Chromosomes A cross-section of part of the nucleus is shown, with the black line representing the nuclear membrane. The green, red, and blue areas indicate three different chromosomes. An interphase chromosome forms a three-dimensional meandering and invaginated territory of (partly) condensed chromatin from which decondensed chromatin fibers extend. Two different modes of organization between neighboring chromosomes are indicated; one on the right between the red and blue chromosomes and the other on the left between the green and the red chromosomes. Domains from the red and blue chromosomes are either separated by interchromatin space or are in touch with no, or little, intermingling of chromatin. By contrast, domains from the green and red chromosomes overlap and their colocalization is indicated in yellow. This figure is based on [ 10].

Understanding which of these models is correct requires a geographical survey of the chromosomes in the cell nucleus by microscopy. The quality of the information with regard to spatial distribution of the components in the nucleus is determined in the first instance by the resolution of the microscope, which is on the order of several hundred nanometers for visual light microscopy and several nanometers for electron microscopy. In general, confocal light microscopy is used for a global overview of the three-dimensional organization of structures in the nucleus, while electron microscopy is useful for detailed two-dimensional views of a small nuclear area. In addition to the type of microscopy employed, the resolution and quality of data obtained are also influenced by changes in the organization of the nuclear components that may occur during preparation of the sample.

Fluorescently labeled DNA sequence-specific probes can be used to selectively “paint” individual chromosomes [ 6]. However, fluorescence in situ hybridization (FISH) requires aggressive treatment of the cells that can often lead to a severe disruption of existing morphology. Nevertheless, the first images produced in the 1980s of interphase chromosomes in the cell nucleus obtained by chromosome painting represent a milestone in cell biology [ 7, 8]. They showed chromosomes occupying distinct territories inside the mammalian cell nucleus, making it obvious that chromosomes in the nucleus are not mixed like spaghetti in a bowl. Close-up pictures showed territories outlined by ruffled borders, leading directly to the question of how chromosomes are arranged at their borders with respect to the surrounding chromosomes [ 6]. Recent developments in multi-color FISH have produced distinct fluorochrome combinations for all 24 chromosome types, making it possible to individually image all the chromosomes in a single cell nucleus [ 1, 6].

Analysis of nuclei stained with multi-color FISH has, however, not yet provided detailed information on the interface between chromosome territories. Morphological degradation, rather than the optical limits of confocal microscopy, limits the resolution of these experiments. Instead, replication labeling has effectively overcome this methodological shortcoming. In this procedure, living cells are incubated during one cell cycle with halogenated or fluorescently labeled nucleosides, which paint all the chromosomes in the replicating cell nuclei. At mitosis, the replicated genome is divided and the painted chromosomes are segregated and distributed between the daughter cells. In the following cell cycles chromosome segregation is repeated and the few painted chromosomes that are transmitted to the (great-) granddaughter cells can be observed clearly against the background of an unlabeled nucleus [ 9]. As nuclear morphology is relatively well preserved, replication labeling provides detailed images of chromosome territory borders. And when subsequently enhanced with gold particles, these labeled nucleosides can be seen under an electron microscope. Ultra-thin sections through single cells showed adjacent condensed chromosome domains separated by interchromatin space at some sites, but also in close contact without interchromatin space at other sites [ 10]. Replication labeling can be repeated with two halogenated nucleosides during two subsequent cell cycles to produce chromosome domains in two colors. Confocal images revealed fiber-like colored chromatin structures embedded in chromosome territories of the other color. Even so, little color overlap was observed in the individual fibers of the chromosome territories [ 11].

In none of these studies were interactions between differently labeled chromosome subdomains or territories analyzed at the ultra-structural level. Yet many fundamental processes influenced by chromosome dynamics, such as transcription and DNA damage repair, are influenced by interactions at that level. To achieve high-resolution detection of distinctly stained chromosomes in nuclei, Miguel Branco and Ana Pombo developed an interesting combination of high-resolution microscopy and accurate multi-color FISH, which they report in this issue of *PLoS Biology* [ 12]. By anchoring ultra-thin sections on glass slides, they limited distortion of the spatial organization of the chromatin that could result from the aggressive FISH procedure. And by combining the dense signal coverage that is produced by fluorescence labeling with the strong gain in vertical optical resolution that is obtained by using ultra-thin sections, they were able to detect fine structural details. From their results, they suggest that adjacent chromosomes display a significant extent of intermingling, thereby challenging cell nucleus models that postulate chromosomes in specific territories separated by interchromatin domain spaces.

The newly reported findings provide an alternative perspective of the organization of chromosome territories in the nucleus. The authors propose that in human lymphocytes, on average, more than 40% of each chromosome is intermingled with the rest of the genome. They further report that this intermingling may have biological effects. By comparing their data on chromosome intermingling with published data on radiation-induced chromosome aberrations in lymphocytes, Branco and Pombo found a highly significant correlation between the extent of intermingling and translocation frequency of certain pairs of chromosomes. Furthermore, they also observed that a general inhibition of transcription induces significant changes in overlap between certain chromosome pairs, which is interpreted as evidence for a relationship between nuclear organization, or architecture, and function.

In addition to fluorescence micrographs, Branco and Pombo have produced electron micrographs of regions where chromosome territories are in contact [ 12]. These high-resolution images show condensed bundles containing chromatin from different chromosomes, suggesting that chromatin fibers of more than one chromosome can come in intimate contact during intermingling. The nucleus is not a static entity, and it seems evident that chromatin mobility should affect chromosome intermingling. Chromatin movement has been observed in the context of several nuclear processes. In cellular division, chromosomes decondense during the formation of daughter nuclei. During interphase, chromatin domains can move randomly within a range of a micrometer [ 13]. This could contribute to chromosome intermingling at chromosome borders. Moreover, large-scale chromatin domains can unfold during gene activation and move over distances of more than a micrometer [ 14], resulting in colocalization of distal genes during their transcription [ 2, 15]. In addition, movement of chromosome domains has also been inferred during DNA double-strand break repair [ 16].

A fascinating question is raised by this new view of chromosomes: how does intermingling and its reversal, disentanglement, take place locally in the interphase nucleus? It is evident that this contact is interrupted when chromosomes condense during mitosis and become separate entities [ 17]. The high-resolution techniques applied in the current study should make it possible to assess identity and concentrations of chromatin proteins in the intermingling regions. Using molecular tools or drugs to change the abundance of proteins involved, one may discover what takes place at the molecular level during the mixing and unmixing of chromatin. We can hope that further methodological innovation will one day allow future studies to be taken to the living cell, to shed light on the dynamic aspects of these tantalizing chromosomal interactions.

## Abbreviation

FISH: fluorescence in situ hybridization
